# Supplementary material for: Non-invasive brain stimulation paradigms in treatment of alcohol use disorder: Systematic review and network meta-analysis protocol
Source: PLoS One. 2025 Oct 7;20(10):e0332857. doi: 10.1371/journal.pone.0332857 (PMC12503284; doi:10.1371/journal.pone.0332857)
Supplement: S1 File — (DOCX) [file pone.0332857.s001.docx]

**S1 File. Search strategies**

Database:

**PubMed**

Date of search: 28/10/24

Results: 154

("transcranial magnetic stimulation"[MeSH Terms] OR "transcranial magnetic stimulation"[All Fields] OR "rTMS"[All Fields] OR "TMS"[All Fields] OR "theta burst stimulation"[All Fields] OR "iTBS"[All Fields] OR "Transcranial Direct Current Stimulation"[Mesh] OR "Transcranial Direct Current Stimulation"[All Fields] OR "tDCS"[All Fields] OR "dTMS"[All Fields] OR "brain stimulation"[All Fields] OR "electrostimulation"[All Fields] OR "NIBS"[All Fields] OR "neuromodulation"[All Fields] OR "tFUS"[All Fields] OR "TPS"[All Fields] OR "pulse stimulation"[All Fields]) AND ("Alcohol drinking"[Mesh] OR "Alcohol drinking"[All Fields] OR "Alcoholism"[Mesh] OR "Alcoholism"[All Fields] OR "alcohol"[All Fields] OR "alcohol use disorder"[All Fields] OR "drinking"[All Fields]) AND ((("randomized controlled trial"[pt] OR "controlled clinical trial"[pt] OR "randomized"[tiab] OR "placebo"[tiab]) OR ("clinical trials as topic" [mesh:noexp]) OR (randomly [tiab] OR trial [ti])) NOT (animals [mh] NOT humans [mh]))

Database

**The Cochrane Library**

Date of search: 28/10/24

Results: 685

Search strategy:

#1 MeSH descriptor: [Transcranial Magnetic Stimulation] explode all trees 2698

#2 transcranial magnetic stimulation 8859

#3 rTMS 4841

#4 TMS 7554

#5 theta burst stimulation 1500

#6 iTBS 922

#7 MeSH descriptor: [Transcranial Direct Current Stimulation] explode all trees 2047

#8 Transcranial Direct Current Stimulation 6920

#9 tDCS 5945

#10 dTMS 153

#11 brain stimulation 15550

#12 electrostimulation 2709

#13 NIBS 309

#14 neuromodulation 4041

#15 tFUS 26

#16 pulse stimulation 3419

#17 TPS 780

#18 MeSH descriptor: [Alcoholism] explode all trees 5163

#19 MeSH descriptor: [Drinking] explode all trees 762

#20 Alcohol drinking 10799

#21 Alcoholism 10022

#22 Alcohol 36690

#23 Alcohol use disorder 5618

#24 drinking 16323

#25 #1 OR #2 OR #3 OR #4 OR #5 OR #6 OR #7 OR #8 OR #9 OR #10 OR #11 OR #12 OR #13 OR #14 OR #15 OR #16 OR #17 31117

#26 #18 OR #19 OR #20 OR #21 OR #22 OR #23 OR #24 43675

#27 #25 AND #26 816

Database

**Embase**

Date of search: 28/10/24

Results: 693

Search strategy:

('brain stimulation'/exp OR 'brain stimulation' OR (('brain'/exp OR brain) AND ('stimulation'/exp OR stimulation)) OR 'electrostimulation'/exp OR 'electrotherapy'/exp OR 'non invasive brain stimulation'/exp OR nibs OR 'transcranial magnetic stimulation'/exp OR 'tms'/exp OR rtms OR dtms OR 'theta burst stimulation'/exp OR 'theta burst transcranial magnetic stimulation'/exp OR 'iTBS' OR 'transcranial direct current stimulation'/exp OR 'neuromodulation'/exp OR 'tFUS'/exp OR 'TPS'/exp OR 'pulse stimulation'/exp) AND ('alcoholism'/exp OR 'alcohol'/exp OR 'drinking'/exp) AND *(*'adaptive clinical trial (topic)'/de OR 'adaptive clinical trial'/de OR 'clinical trial (topic)'/de OR 'clinical trial'/de OR 'controlled clinical trial (topic)'/de OR 'controlled clinical trial'/de OR 'double blind procedure'/de OR 'early termination of clinical trial'/de OR 'equivalence trial (topic)'/de OR 'equivalence trial'/de OR 'intention to treat analysis'/de OR 'multicenter study (topic)'/de OR 'multicenter study'/de OR 'non-inferiority trial'/de OR 'phase 1 clinical trial (topic)'/de OR 'phase 1 clinical trial'/de OR 'phase 2 clinical trial (topic)'/de OR 'phase 2 clinical trial'/de OR 'phase 3 clinical trial (topic)'/de OR 'phase 3 clinical trial'/de OR 'phase 4 clinical trial (topic)'/de OR 'phase 4 clinical trial'/de OR 'pragmatic trial'/de OR 'randomized controlled trial (topic)'/de OR 'randomized controlled trial'/de OR 'superiority trial'/de OR 'multicenter study':ti,ab,kw OR 'phase I':ti,ab,kw OR 'phase II':ti,ab,kw OR 'phase III':ti,ab,kw OR 'phase IV':ti,ab,kw OR 'phase 1':ti,ab,kw OR 'phase 2':ti,ab,kw OR 'phase 3':ti,ab,kw OR 'phase 4':ti,ab,kw OR ((randomised OR randomized) NEAR/7 trial*) OR (controlled NEAR/3 trial*) OR (clinical NEAR/2 trial*) OR ((single:ti,ab,kw OR doubl*:ti,ab,kw OR tripl*:ti,ab,kw OR treb*:ti,ab,kw) and (blind*:ti,ab,kw OR mask*:ti,ab,kw)) OR '4 arm':ti,ab,kw OR 'four arm':ti,ab,kw)

Database

**PsycINFO (via EBSCO)**

Date of search: 28/10/24

Results: 510

transcranial magnetic stimulation or tms or rtms or tdcs or transcranial direct current stimulation or non invasive brain stimulation or tps or pulse stimulation or tfus or theta burst stimulation or itbs or electrostimulation or neuromodulation or dtms

AND alcoholism or alcohol dependence or alcohol abuse or alcoholic or alcohol addiction or drinking

Database

**Clinicaltrials.gov**

Date of search: 28/10/24

Results: 49

Search strategy:

(Non-invasive brain stimulation) OR (Transcranial Magnetic Stimulation) OR (TMS) OR (Transcranial Direct Current Stimulation) OR (tDCS) OR (theta-burst stimulation) OR (iTBS) OR (neuromodulation) OR (TPS) OR (pulse stimulation) OR (tFUS)| Alcohol Use Disorder

Database

**WHO ICTRP**

Date of search: 28/10/24

Results: 135

(“transcranial magnetic stimulation” OR “rTMS” OR “TMS” OR “theta burst stimulation” OR “iTBS” OR “Transcranial Direct Current Stimulation” OR “tDCS” OR “dTMS” OR “brain stimulation” OR “electrostimulation” OR “neuromodulation” OR “non invasive brain stimulation” OR “NIBS” OR “pulse stimulation” OR “TPS” OR “tFUS”) AND (“Alcoholism” OR “Alcohol use disorder” OR “alcohol” OR “drinking”)

Database

**EU Clinical Trials**

Date of search: 28/10/24

Results: 5

Search strategy:

((transcranial magnetic stimulation) OR (rTMS) OR (TMS) OR (theta burst stimulation) OR (iTBS) OR (Transcranial Direct Current Stimulation) OR (tDCS) OR (dTMS) OR (brain stimulation) OR (electrostimulation) OR (neuromodulation) OR (non invasive brain stimulation) OR (NIBS) OR (pulse stimulation) OR (TPS) OR (tFUS)) AND ((Alcoholism) OR (Alcohol use disorder) OR (alcohol) OR (drinking))
