# Supplementary material for: Non-invasive brain stimulation paradigms in treatment of alcohol use disorder: Systematic review and network meta-analysis protocol
Source: PLoS One. 2025 Oct 7;20(10):e0332857. doi: 10.1371/journal.pone.0332857 (PMC12503284; doi:10.1371/journal.pone.0332857)
Supplement: S2 File — (DOCX) [file pone.0332857.s002.docx]

| **First author, year** | **Country** | **Design** | **AUD characteristics** | **Population (N, % female), mean age** | **Intervention (type, characteristics)** | **Control (characteristics)** | **Timeline** | **Outcomes and scales** | **Results (interpretation)** |
| --- | --- | --- | --- | --- | --- | --- | --- | --- | --- |
|  |  |  |  |  |  |  |  |  |  |
|  |  |  |  |  |  |  |  |  |  |
|  |  |  |  |  |  |  |  |  |  |
|  |  |  |  |  |  |  |  |  |  |
|  |  |  |  |  |  |  |  |  |  |

**S2 File. Extraction form**
