# Supplementary figures and images for: Non-invasive brain stimulation paradigms in treatment of alcohol use disorder: Systematic review and network meta-analysis protocol
Source: PLoS One. 2025 Oct 7;20(10):e0332857. doi: 10.1371/journal.pone.0332857 (PMC12503284; doi:10.1371/journal.pone.0332857)

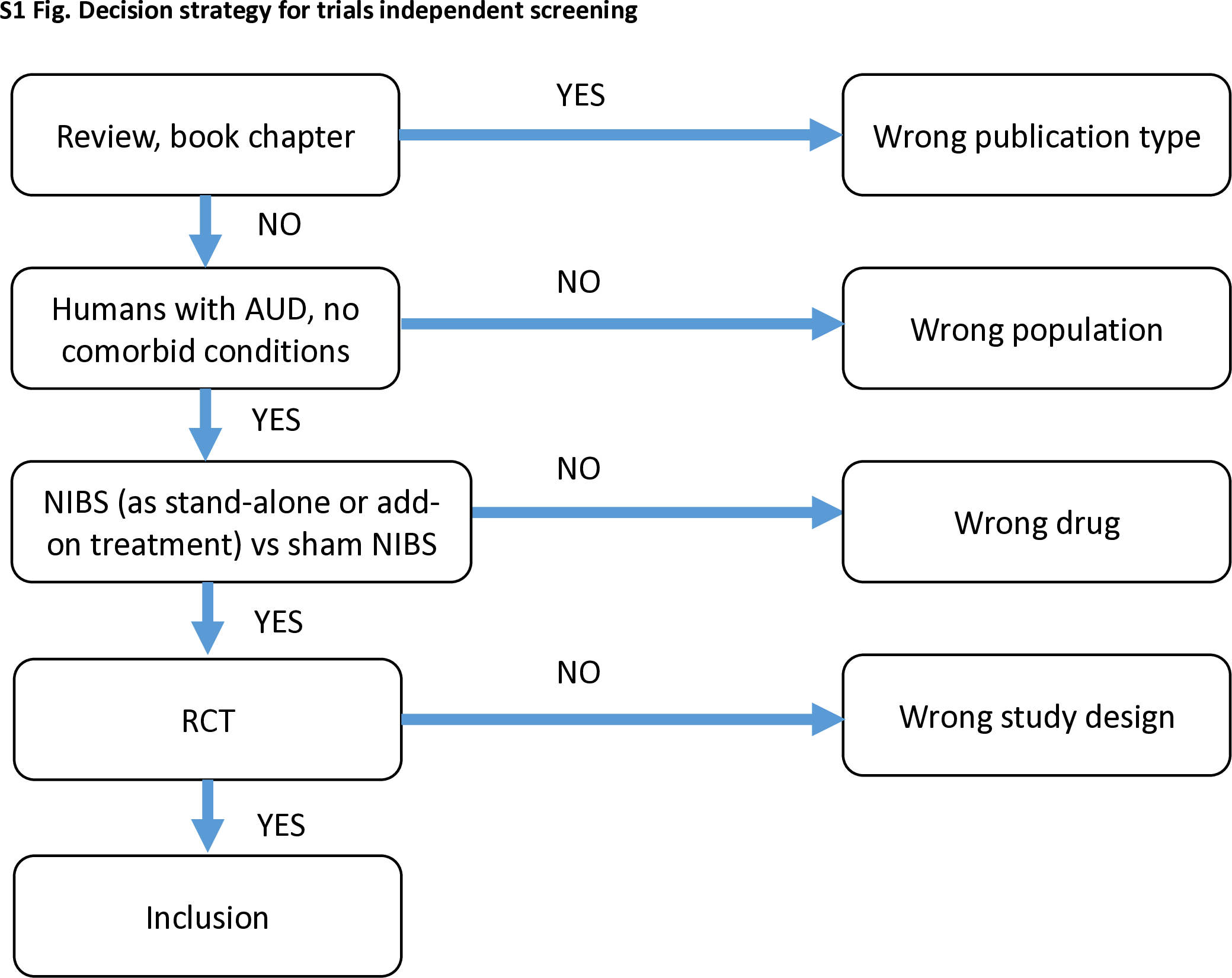

Supplement: S1 Fig — (TIF) [file pone.0332857.s003.tif]
