# Supplementary material for: Non-invasive brain stimulation paradigms in treatment of alcohol use disorder: Systematic review and network meta-analysis protocol
Source: PLoS One. 2025 Oct 7;20(10):e0332857. doi: 10.1371/journal.pone.0332857 (PMC12503284; doi:10.1371/journal.pone.0332857)
Supplement: Attachment — Submitted filename: Response to Reviewers.docx [file pone.0332857.s005.docx]

PONE-D-24-54144

Non-invasive brain stimulation paradigms in treatment of alcohol use disorder: systematic review and network meta-analysis protocol

**PLOS ONE**

**Dear Academic Editor, Dear Reviewers,**

Thank you for your careful attention and valuable comments on our manuscript.

Please find our responses below.

**General Comments**

1. We made the necessary modifications to ensure that the manuscript adheres to PLOS ONE’s style requirements.
2. We placed the ethics statement at the beginning of the Methods section.
3. For image formatting, we unfortunately experienced difficulties connecting to the https://pacev2.apexcovantage.com/ server. How can we ensure that our figure adheres to PLOS ONE’s requirements without access to this software?

**Reviewer #1:**

1 & 2. We added a Discussion section and addressed the limitation concerning the use of Psychological Index Terms in the PsycINFO search strategy in it (L236-260).

**Reviewer #2:**

1. We added a scientific rationale for this decision and discussed the limitation in the Discussion section (L240-249).
2. We revised the Methods section to clarify how the nodes of the network meta-analysis (NMA) were defined (L201-206).
3. We provided additional detail on the length of follow-up and the definitions of relapse, and we discussed these points in the Discussion section (L111-114, L120-124).
4. We added a paragraph describing the approach to exploring heterogeneity, including pre-defined sensitivity analyses (L218-223).
5. We added a section on the confidence in the overall evidence and explained the use of the CINeMA framework, which is based on the GRADE approach with specific adaptations for NMA methodology (L230-235).
