# Supplementary material for: Non-invasive brain stimulation paradigms in treatment of alcohol use disorder: Systematic review and network meta-analysis protocol
Source: PLoS One. 2025 Oct 7;20(10):e0332857. doi: 10.1371/journal.pone.0332857 (PMC12503284; doi:10.1371/journal.pone.0332857)
Supplement: Attachment — Submitted filename: Response to Reviewers2.docx [file pone.0332857.s006.docx]

PONE-D-24-54144

Non-invasive brain stimulation paradigms in treatment of alcohol use disorder: systematic review and network meta-analysis protocol

**PLOS ONE**

**Dear Academic Editor, Dear Reviewers,**

We thank you for your positive assessment of our revised manuscript and for the valuable advice to further enhance its methodological transparency and reproducibility. Please find our responses below:

1. We provided additional details on the expected definitions of relapse and the proposed methods for harmonization (L118–125).
2. We included information on how the *netmeta* package accounts for the correlation of pairwise comparisons in multi-arm studies (L197–198).
3. We briefly explained how the RoB 2 assessments will be incorporated into the CINeMA framework.

We sincerely appreciate your continued consideration of our manuscript and the valuable comments that have helped improve it.

Dr Anastasia DEMINA
